# Methotrexate-Induced Stroke-Like Encephalopathy: Beware the Stroke Mimic

**DOI:** 10.5334/jbsr.2935

**Published:** 2022-10-31

**Authors:** Adam Benkirane, Nicolas Mulquin, Frédéric London

**Affiliations:** 1 CHU UCL Namur site Godinne, BE

**Keywords:** methotrexate, stroke-like encephalopathy, magnetic resonance imaging

## Abstract

We report a case of methotrexate (MTX)-induced stroke-like encephalopathy in an 18-year-old woman, with acute lymphoblastic leukemia, who developed a sudden neurological deficit mimicking a cerebrovascular event. Bain MRI showed hyperintensities on diffusion-weighted-imaging (DWI) with matching apparent diffusion coefficient hypointensities, which also represent the commonest MRI findings in acute cerebral infarction. DWI changes spared the cerebral cortex and did not respect vascular territories, supporting a non-vascular mechanism. MRI plays a crucial role in the diagnostic work-up and is essential to avoid unnecessary intervention such as thrombolytic therapy.

**Teaching Point:** Methotrexate-induced stroke like neurotoxicity should be considered in patients treated with methotrexate and presenting with a stroke-like clinical picture and radiological findings consistent with acute cerebral infarction.

## Introduction

Methotrexate (MTX) is a folic-acid antagonist commonly used to treat a range of autoimmune diseases and malignancies. Rarely, MTX neurotoxicity may present as stroke-like onset, causing diagnostic dilemma. We herein present a novel observation of MTX-induced stroke-like encephalopathy in a young patient with acute lymphoblastic leukemia (ALL) and who developed a sudden neurological deficit mimicking a cerebrovascular event.

## Case History

**Figure 1:**
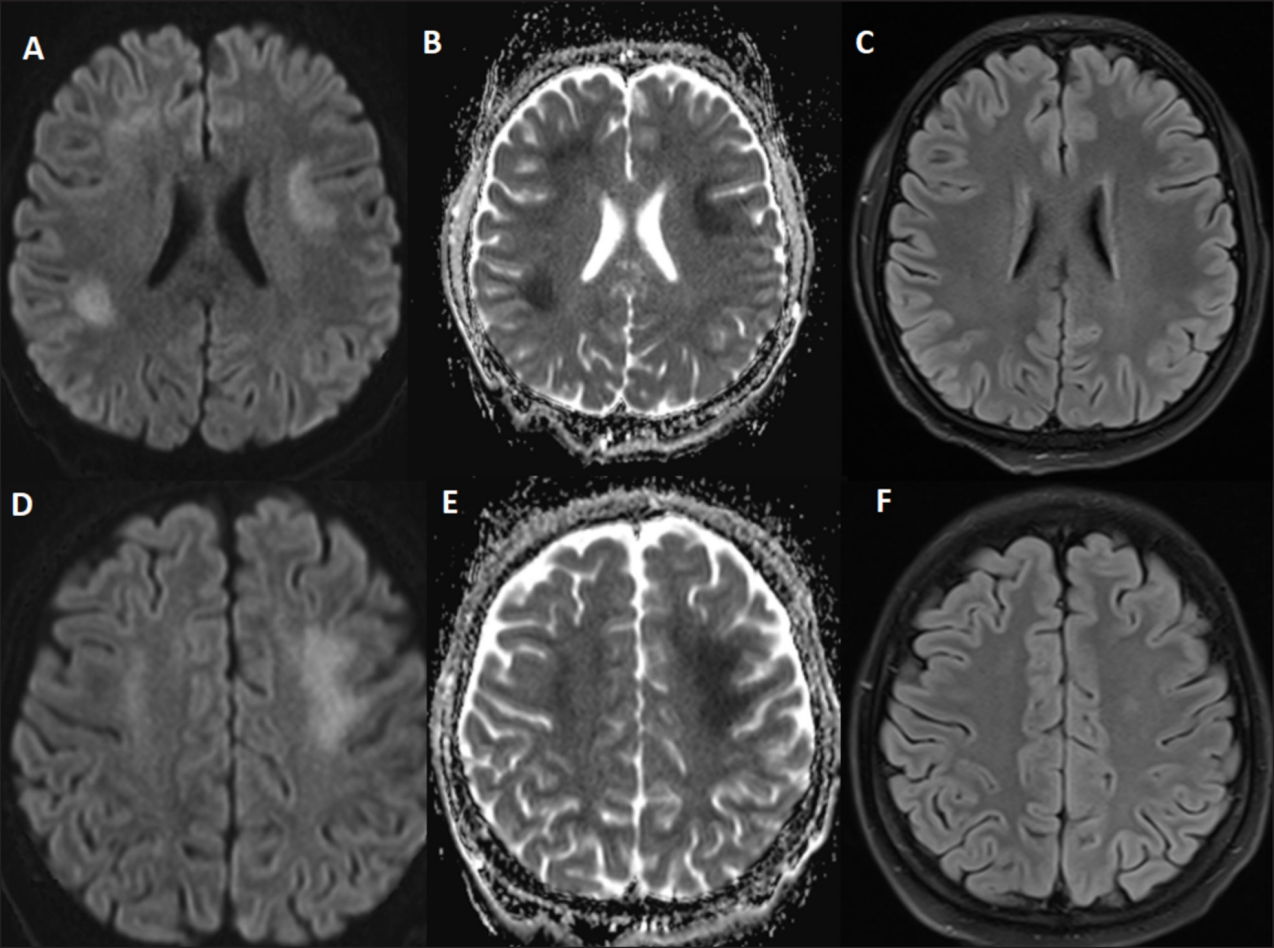
Brain MRI showing bilateral hyperintense diffusion-weighted imaging signals **(A, D)** and decreased apparent diffusion coefficient **(B, E)** in the white matter of the frontal and parietal lobes. Axial fluid attenuated inversion recovery image was normal **(C, F)**.

**Figure 2:**
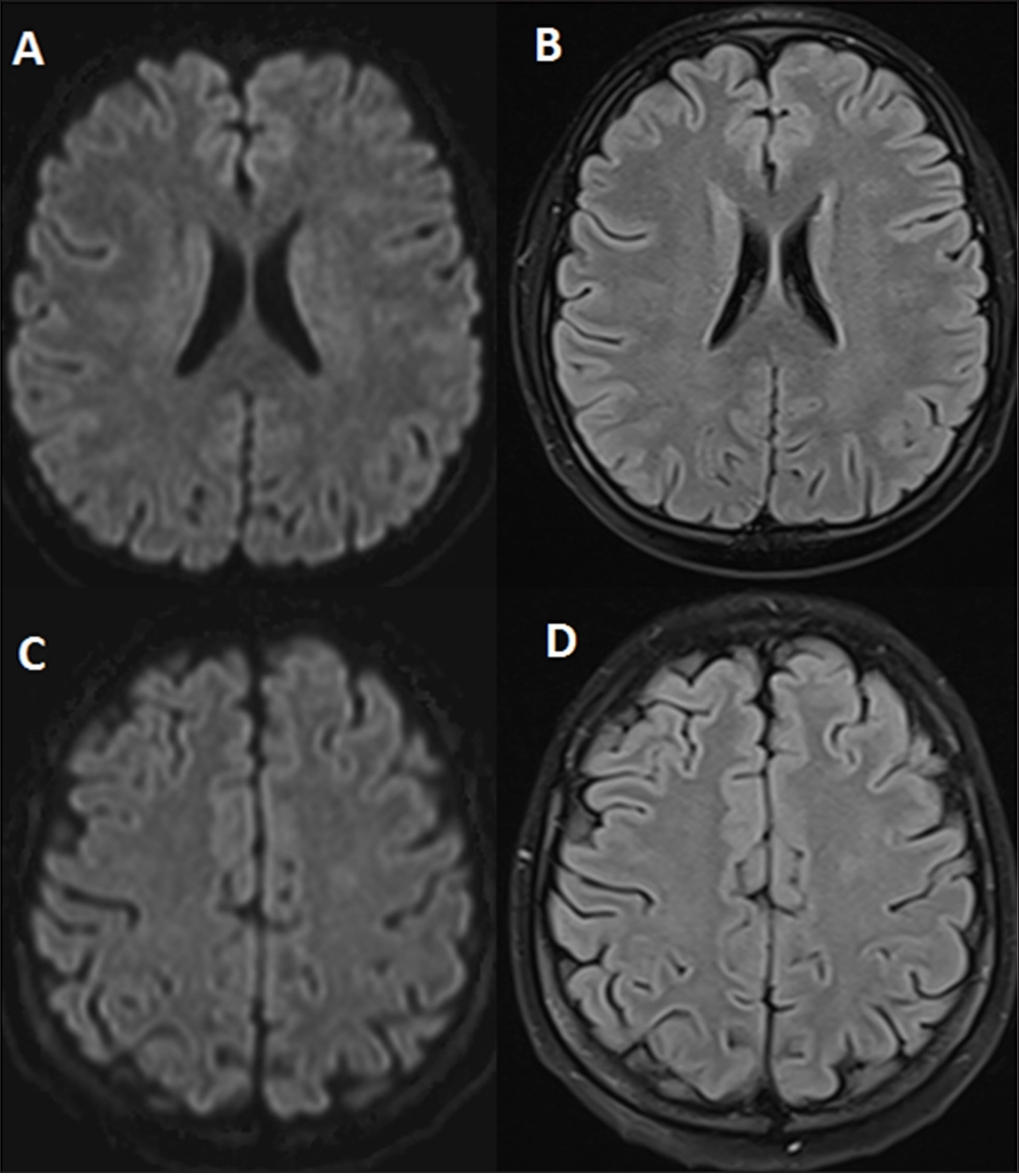
Follow-up brain MRI at four weeks showing complete resolution of radiological abnormalities (**A, C:** axial diffusion-weighted imaging; **B, D:** Axial fluid attenuated inversion recovery images).

An 18-year-old woman was diagnosed with B-cell ALL in August 2019. The patient was included in the GRAALL 2014 study (ClinicalTrials.gov Identifier: NCT02617004). Morphological remission was achieved after induction therapy. In December 2019, three days after completing the fifth consolidation cycle, consisting of vincristine (total dose 2 mg), 6-mercaptopurine (60 mg/m^2^), intravenous MTX (1g/m^2^), triple intrathecal therapy (MTX 15 mg, cytarabine 40 mg and hydrocortisone 40 mg), she presented abrupt onset of aphasia and right facial weakness at 11:30 a.m. She was admitted to the Emergency Department at 12:40 p.m. Initial National Institutes of Health Stroke Scale (NIHSS) score was 8. Blood sugar was normal; vital signs were unremarkable. Due to the sudden onset of symptoms, stroke was first considered. An urgent brain magnetic resonance imaging (MRI), performed within 2h of symptom onset, showed bilateral diffusion-weighted imaging (DWI) hyperintensities involving the subcortical white matter (Figure 1). There were also matching hypointensities on the apparent diffusion coefficient (ADC) map, but no signal change on fluid-attenuated inversion recovery (FLAIR) was observed (Figure 1). Angiography showed no evidence of vasospasm. DWI changes sparing the cerebral cortex and not respecting vascular territories supported a non-vascular mechanism. Consequently, the patient was excluded for thrombolytic therapy. Cerebrospinal fluid analysis, performed to rule out opportunist infection, was unremarkable; cultures and polymerase chain reactions for various neurotropic viruses were all negative. Based on the radiological and laboratory findings, and the temporal relationship with administration of MTX, MTX-induced neurotoxicity was therefore considered. Conservative therapy was administered. The patient had complete resolution of all the symptoms within 24 hours. Follow-up MRI at four weeks showed complete resolution of radiological abnormalities (Figure 2). After consulting the GRAALL scientific board for guidance regarding MTX continuation, it was decided to no longer administer MTX to the patient. No neurological manifestations occurred during a 30-month follow-up.

## Discussion

Stroke-like encephalopathy is a rare MTX neurotoxicity, occurring days to weeks after MTX administration, and presenting with a sudden onset of focal neurological deficits [1]. In a large randomized trial on ALL, 31 patients had a ‘stroke-like syndrome’ caused by intrathecal MTX administration [2]. In another prospective study focusing on the neurotoxicity of MTX in 369 children with ALL, stroke-like symptoms were only reported in 6 patients [3]. Unlike patterns of stroke on MRI, typical MRI findings in acute symptomatic MTX-induced stroke like encephalopathy demonstrate transient, multifocal and reversible restricted diffusion in the subcortical and periventricular white matter, without initial FLAIR signal abnormalities [1]. In stroke, the MRI abnormalities are related to excitotoxic effects of excessive release of glutamate in the synaptic cleft and disruption of glutamate reuptake, ultimately resulting in irreversible cytotoxic edema and neuronal death. In opposition, the mechanism plausibly involved in the pathophysiology of MTX-stroke like encephalopathy is an intramyelinic edema, which refers to a non-neurotoxic edema. In this type of edema, the water molecules are unable to shift to other extracellular spaces, but the reuptake of glutamate is maintained by the myelin sheath and the astrocytes, therefore protecting the axon from death [4]. This explains the transiently restricted diffusion lesions and the absence of FLAIR abnormalities, as observed in our patient. In the peculiar situation of acute neurological deficits in patients receiving MTX, MRI therefore plays a crucial role in the diagnostic work-up and outperforms computed tomography. It should be the first choice for imaging.

Multiple underlying mechanisms are possibly involved, including (i) a direct toxicity of homocysteine on the vascular endothelium, (ii) an increased excitatory effects on N-methyl-D-aspartate receptors by homocysteine-derived metabolites, (iii) alterations of adenosine metabolism, (iiii) a chronic folate depletion in brain tissue, and (iiiii) a direct neuronal damage by MTX [5]. In most reported cases, re-exposure to intrathecal MTX administration was not associated with recurrence of neurotoxicity, but any treatment decision must include a careful weighing of potential risks of relapse against anti-tumor benefits [1,2].

## Conclusion

Our observation underlines the importance of considering MTX-induced stroke like neurotoxicity in patients treated with MTX and presenting with a stroke-like clinical picture. Emergency MRI with DWI is the mainstay of imaging to discriminate this stroke-mimicking condition from a real stroke.
